# Typology and implications of verified attacks on health care in Ukraine in the first 18 months of war

**DOI:** 10.1371/journal.pgph.0003064

**Published:** 2024-05-23

**Authors:** Hyo-Jeong Kim, Emanuele Bruni, Galyna Gorodetska, Rafael Van den Bergh, Lamia Bezer, Sergiy Artykutsa, Noémie Andriamiseza, Jarno Habicht

**Affiliations:** 1 Attacks on Health Care Initiative, WHO, Geneva, Switzerland; 2 WHO Country Office for Ukraine, Kyiv, Ukraine; Royal Infirmary of Edinburgh, UNITED KINGDOM

## Abstract

Attacks on health care are part of the spectrum of threats that health care endures during conflict. Protecting health care services against attacks depends on understanding the nature and types of attacks that occur during conflict. The World Health Organisation has implemented the Surveillance System for Attacks on Health Care (SSA) in Ukraine since 2020, and the system has continued to monitor and report on attacks on health care during the war in Ukraine. This study aims to analyse the data reported through the SSA for the first 18 months of the war. This paper involves a retrospective, descriptive study based on the analysis of publicly available SSA data of all incidents of attacks on health care in Ukraine reported through the SSA between February 24^th^ 2022 and August 24^th^ 2023. Out of the 1503 verified attacks, 37% occurred in the initial six weeks of the war. Attacks involving violence with heavy weapons were among the most common incidents reported (83%). The reported attacks were associated with a total of 113 deaths and 211 injuries among health care workers and patients: 32 (2%) attacks were associated with a death of a health care worker or patient, and 63 (4%) were associated with an injury. Health transports facing attacks had a higher probability of experiencing casualties than other health resources (p<0.0001, RR 3.1, 95%CI 1.9–4.9). In conclusion, the burden of attacks on health care in Ukraine was high and sustained over the course of the first 18 months of the war. Reported casualties were not homogenously distributed among attack incidents, but occurred in a set of high-casualty incidents. Health transports were found to be particularly vulnerable. In addition to continued calls for a cessation of hostilities, prevention, protection, mitigation, and reconstruction strategies are urgently required.

## Background

Attacks on health care are part of the spectrum of threats that health care endures during conflict. They carry a particular weight within this spectrum, as their repercussions are both immediate and long-term and deprive people of essential health services [1]. Consequences of attacks are multidimensional, and include an impact on health care workers (death and injury, mental distress, workforce attrition), patients (death and injury, loss of health care options, fear of attending health care), and health systems (destruction of health infrastructure such health facilities, ambulances, and warehouses; loss of resources; long-term destabilisation and collapse of the health system) [2,3]. Additionally, attacks on health care can represent important violations of international humanitarian law (IHL) and international human rights law (IHRL) [4,5].

Despite multiple appeals and resolutions calling for the protection of health care [6–8], data from the World Health Organization (WHO) Surveillance System for Attacks on Health Care (SSA) shows that attacks on health care continue unabated [3]. Attacks on health care are a global phenomenon, occurring in large-scale, open conflicts, as well as in localised conflicts and even as element of community clashes [2,3]. Approaches for protecting health care services during conflict are predicated on a full understanding of the risks faced by different health resources in relation to the conflict dynamics. Analyses of data from Afghanistan, Somalia and the Democratic Republic indicate the value of in-depth assessments of attack data [9]. However, due to the challenging nature of conducting research in conflict settings, few analyses have been conducted to describe the characteristics and (temporal) patterns of attacks on health care in modern, large-scale conflict environments [10]. Evidence from Syria shows how attacks are closely tied to contextual changes in the conflict, and that different types of health facilities bear a differential risk for e.g. repeat attacks [11,12]. Studies also indicate the relevance of monitoring attacks on health care for assessing overall violence against civilians [11].

The war in Ukraine, which escalated on February 24^th^ 2022, has resulted in substantial civilian casualties, displacement of millions of people, widespread destruction of infrastructure and, as a result, disruption of health service delivery. Overall, the health system of Ukraine has been described as resilient in the face of war [13–15]. Nevertheless, an assessment of access to health services among the Ukrainian population in late 2022 showed that more than half of those who sought health care faced at least one challenge in doing so, mainly related to cost of medicines and treatment, time and transport, and unavailability of services [16]. Such disruptions were in part related to attacks on health care, which have been recorded in Ukraine through the SSA since before the start of the hostilities [3]. While SSA data has been made publicly available, only few in-depth analyses have been conducted to better understand the (temporal) patterns of attacks and their immediate consequences [17–19]. With the aim of generating an evidence base for guidance on developing protection and mitigation measures against attacks on health care, and identifying focus areas for future in-depth investigation and research, we conducted an analysis of the characteristics of the verified attacks on health care in Ukraine reported through the SSA, between February 24^th^ 2022 and August 24^th^ 2023.

## Methods

### Study design

This was a retrospective, descriptive study based on the analysis of publicly available data.

### Setting—Ukrainian health system

Prior to the escalation of violence in February 2022, the health care system of Ukraine was already confronted with a humanitarian crisis, linked to the conflict that started in 2014 [20]. Since 2016, the health care system was in reform, with progress being made focusing on public health, strengthening health financing, and supporting primary health care provision, e-health, and ensuring access to medicines. In 2019, 39% of the population attended outpatient services over the past year and 14% had a hospitalisation episode. The main concern of the population with their health care options was the high cost of medications; among outpatient care users, 21% participated in the “Affordable Medicines” programme, with more than half of the participants in the programme reporting improved access to medicines over the past year [21].

### Attacks on health care—Definitions

Attacks on health care are defined relatively widely by WHO as “any act of verbal or physical violence or obstruction or threat of violence that interferes with the availability, access and delivery of curative and/or preventive health services during emergencies” [1]. Such acts include heavy weapons attacks (such as bombing and shelling) and attacks with individual weapons, but also incidents such as theft/looting of health assets and militarization of health care. The latter is defined as “diversion of or interference with the primary use of civilian (i.e., non-military) health care facilities or transport by state military or paramilitary forces or non-state armed groups” [22], and–in addition to the diversion of resources–represents an important risk for health care, as it can lead to a perceived loss of neutrality of health services, exposing them to further acts of violence.

### Surveillance for attacks on health care by WHO

WHO verifies information of potential attacks through triangulation of different sources and types of information to ascertain whether an incident took place or not. Each incident is assigned a level of certainty based on the strength of the information used in the verification. The report is reviewed by 3 designated persons in WHO and is then published on the public dashboard of the SSA. The reporting, verification, and publication process can range from several days to months, meaning that data on the public dashboard continues to be subject to change. For security reasons, no geographical disaggregation is offered for the public SSA data: data is only reported on at country level. In general, WHO addresses attacks on health care as incidents that compromise access to and delivery of health care, rather than from a medicolegal/IHL perspective. Perpetrators and potential motivations of attacks are for instance not included in the SSA datasets, as investigation is not part of the WHO mandate for surveillance of attacks on health care [23]. A more detailed description of the SSA methodology is available in online WHO resources [22].

### Study period and study population

All incidents of attacks on health care reported through the SSA between February 24^th^ 2022 and August 24^th^ 2023 were included in this study. As the SSA is an incident-based surveillance system, the study population consisted of incidents, which could include multiple attacks occurring at different timepoints on a single health service. The impacted health resources include health facilities, health workers, patients, and health transports, as defined in the SSA methodology [22].

Four distinct phases of the war were identified throughout the study period, to allow assessment of changes in patterns of attacks over time. Interactive maps covering the study period can be accessed through the Institute for the Study of War (ISW) [24]:

- Phase 1: from February 24^th^ to April 7^th^ 2022, covering rapid mobilization of the population in Ukraine and seeing heavy fighting in Kyiv and the northeast of Ukraine, including the start of the siege of Mariupol.
- Phase 2: from April 8^th^ to August 31^st^ 2022, covering a redeployment of Russian forces from the Kyiv area to the Donbas region and hostilities focused on the southeast.
- Phase 3: from September 1^st^ to November 30^th^ 2022, covering a Ukrainian counteroffensive and fighting in the Kharkiv region in the north and Kherson region in the south of Ukraine. From October 10^th^ 2022 onwards, this phase also included widespread strikes on Ukrainian energy infrastructure [25].
- Phase 4: from December 1^st^ 2022 to August 24^th^ 2023, a positional war phase, covering the battles for Soledar and Bakhmut, with heavy losses on both sides. This period also saw the destruction of the Kakhovka Dam, leading to significant infrastructural devastation, population displacement, and likely long-term environmental consequences for the region.

### Sources of data and data collection

All incidents of attacks on health care reported over the study period were extracted from the public SSA repository [3]. Extractions were performed on March 20^th^ 2024; only the publicly available data from the SSA was extracted. It should be highlighted that due to the dynamic and complicated nature of attack notification and verification, additional attacks in the earlier phases of the war may have been added to the public repository after the extraction date and have as such not been included in this analysis. All data was transferred to a dedicated study database for analysis.

### Data analysis

Attack incident data was represented through summary statistics. Associations between attack characteristics were assessed through bivariate analysis using a chi^2^ test, with p-values<0.05 being considered significant. The magnitude of association was expressed using Risk Ratios (RR) and their corresponding 95% confidence intervals (95%CI). Statistical analysis was conducted using EpiData Analysis v.2.2.3 (EpiData Organisation, Odense, Denmark).

### Ethics

As this was a study of routinely collected programme data that is available through a public repository, and as no identifiable information concerning individuals or of specific health facilities was used, no ethics review was sought.

## Results

### Patterns of attacks

**Fig 1:**
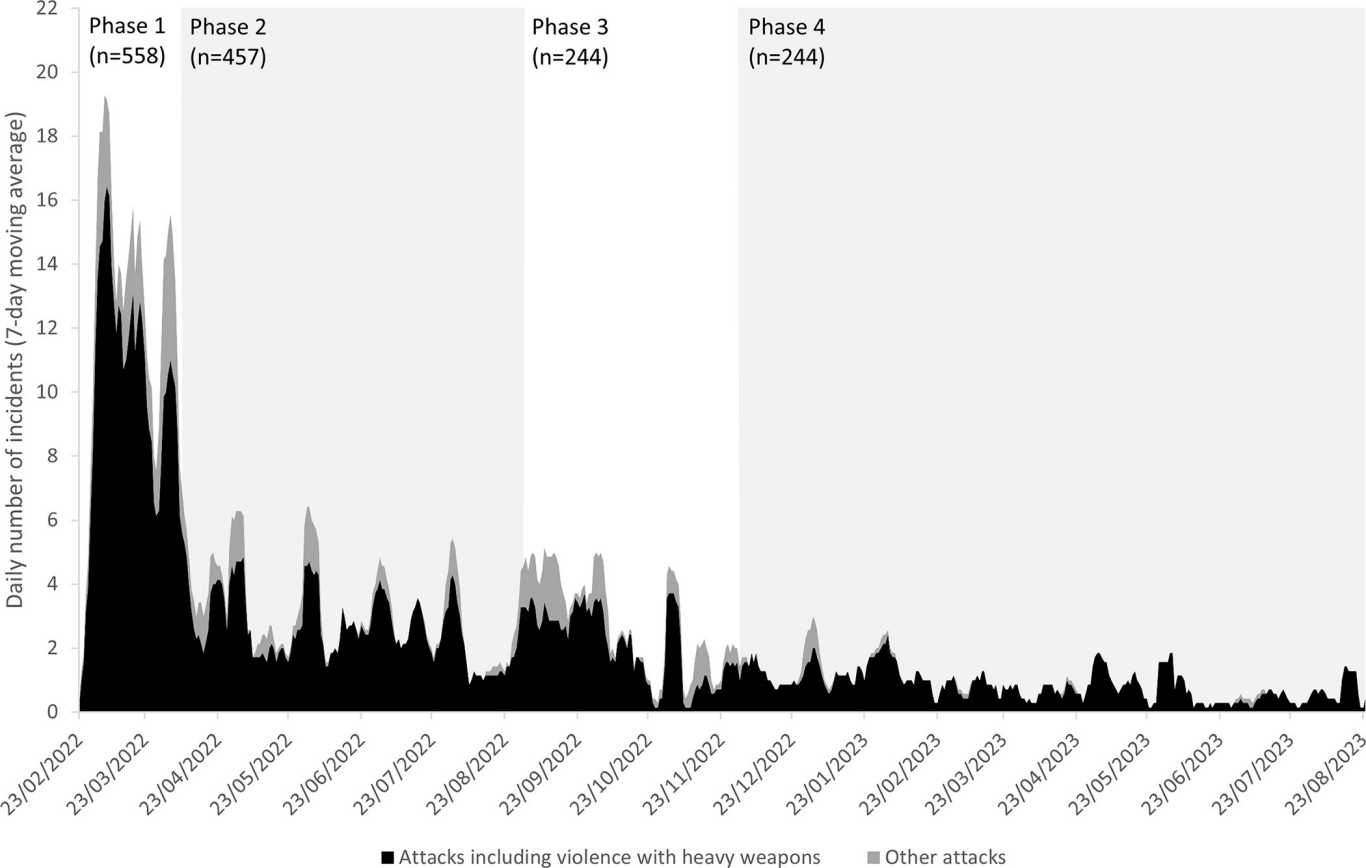
Number of attacks on health care (7-day moving average) reported to WHO’s Surveillance System for Attacks on Health Care (SSA) in Ukraine, February 2022-August 2023.

**Fig 2:**
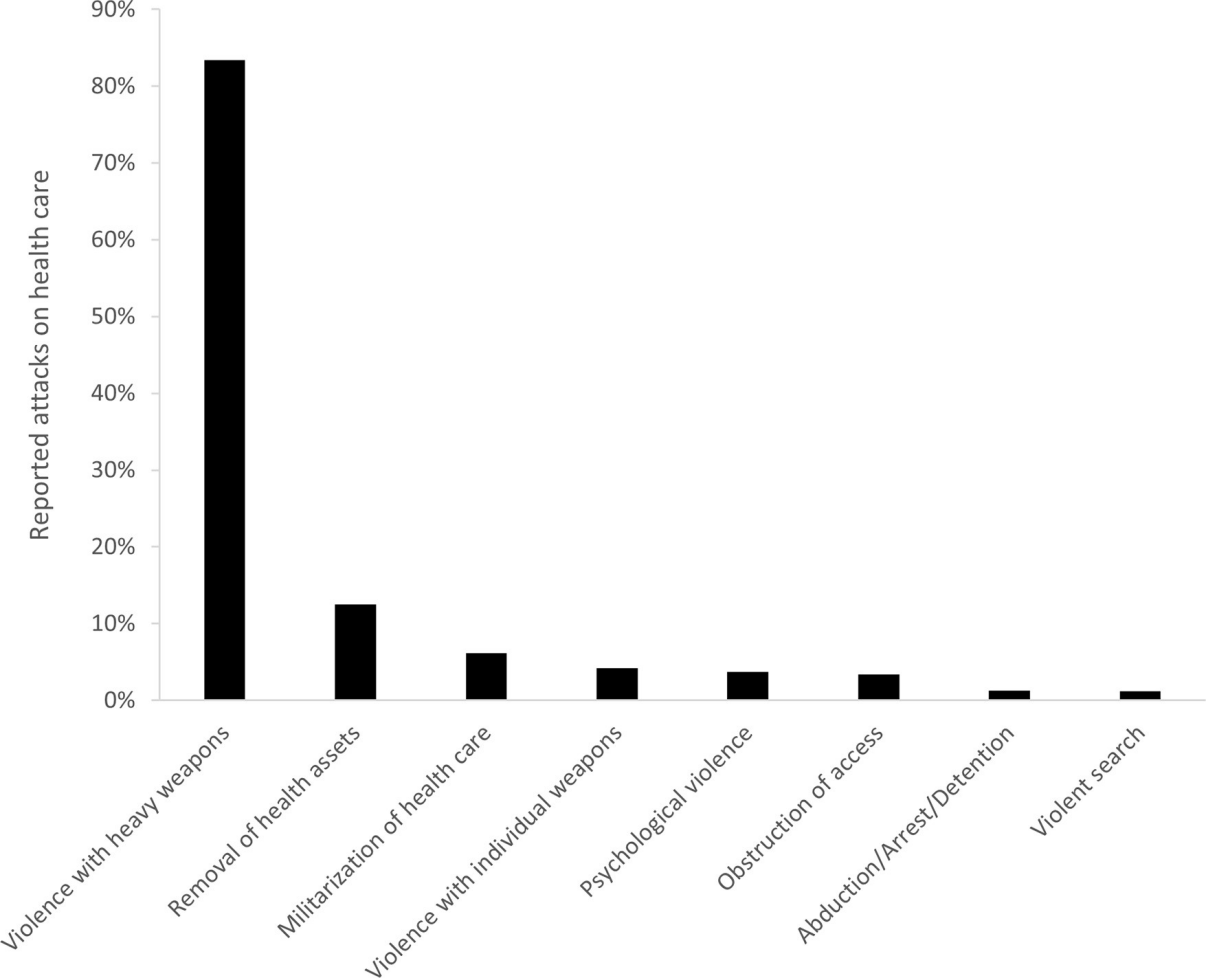
Proportion of attack types out of all attacks on health care (N = 1431) as reported to WHO’s Surveillance System for Attacks on Health Care (SSA) in Ukraine, February 2022-August 2023.

Over the study period, a total of 1503 incidents of attacks were published through the SSA. The timeline of attacks, broken down in attacks involving violence with heavy weapons and other types of attacks, is shown in Fig 1 (using a 7-day moving average): the majority of attacks (37%) took place in phase 1 of the conflict. Types of attacks are indicated in Fig 2: attacks involving violence with heavy weapons were the most common types of incidents (83%), followed by removal of health assets (including theft and looting; 13%) and militarization of health care (6%). Militarization of health care was more likely to be reported in phase 1 (p = 0.001, RR 1.9, 95%CI 1.3–2.8) and phase 3 (p<0.0001, RR 3.0, 95%CI 2.0–4.4) of the war; removal of health assets was more likely to be reported in phase 3 of the war (p<0.0001, RR 2.1, 95%CI 1.6–2.8). The majority of attacks had a single typology (89%): of note, incidents of militarization of health care were significantly more likely to be associated with a multiple typology/other types of attacks (p<0.0001, RR 13.5, 95%CI 10.8–16.7).

### Casualties

The reported attacks were associated with a total of 113 deaths and 211 injuries among health care workers and patients. Strikingly, these casualties were related to a limited set of incidents: 32 (2%) attacks were associated with a death of a health care worker or patient, and 63 (4%) were associated with an injury. One individual attack was associated with 50% of all deaths, and a second individual attack was associated with 17% of all injuries over the study period. Overall, among attacks involving violence with heavy weapons, a rate of 0.8 deaths/10 attacks and 1.6 injuries/10 attacks was observed. Incidents during phase 1 of the war had a higher probability of being associated with deaths (p = 0.009, RR 2.5, 95%CI 1.2–5.0); 76% of all deaths (and 44% of all injuries) occurred in phase 1.

### Affected resources

**Fig 3:**
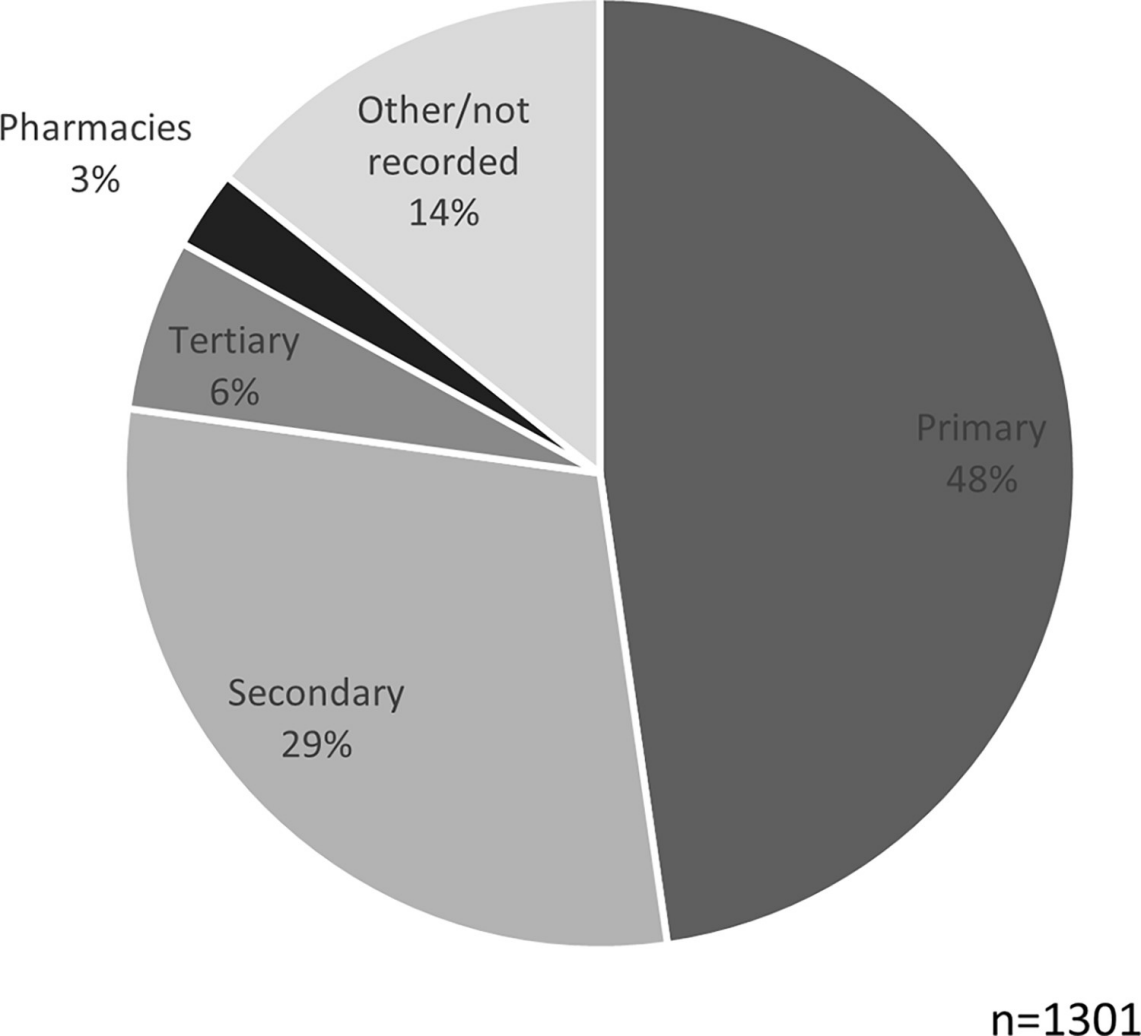
Health facility types affected by attacks; as reported to WHO’s Surveillance System for Attacks on Health Care (SSA) in Ukraine, February 2022-August 2023.

**Fig 4:**
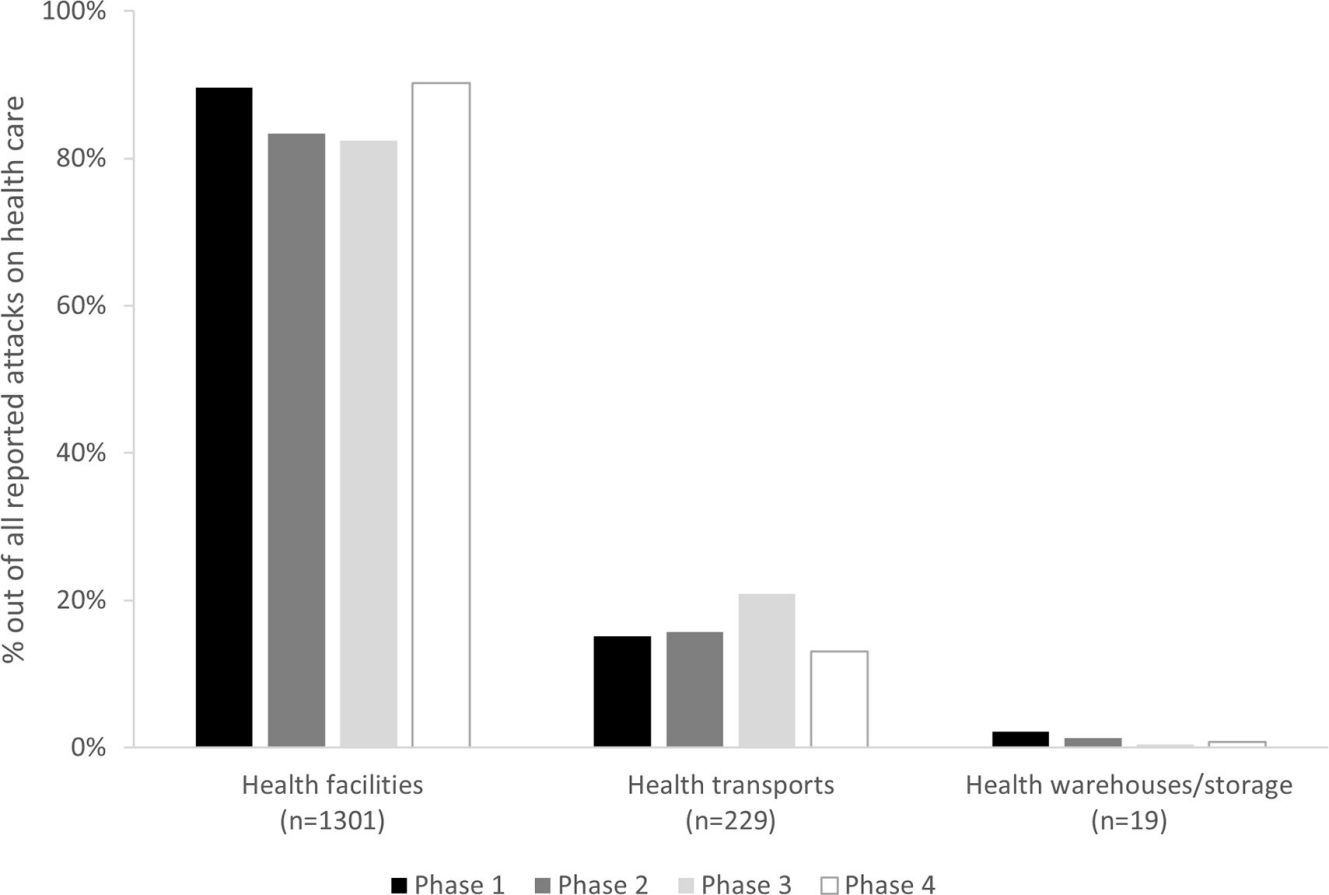
Proportion of affected health infrastructure out of all reported attacks per time period; as reported to WHO’s Surveillance System for Attacks on Health Care (SSA) in Ukraine, February 2022-August 2023.

Attacks against three types of health infrastructural resources were assessed: health facilities, health transports, and health warehouses/storage facilities [22]. Attacks affecting health facilities were the most common (87%), followed by health transports (16%) and health warehouses/storage facilities (1%). Among the affected health facilities, the majority were primary health care facilities (Fig 3). Patterns of attacked health resources persisted through the different phases of the conflict: attacks on health transports and health storage facilities decreased slightly over time, while attacks on health facilities remained predominant throughout (Fig 4).

**Fig 5:**
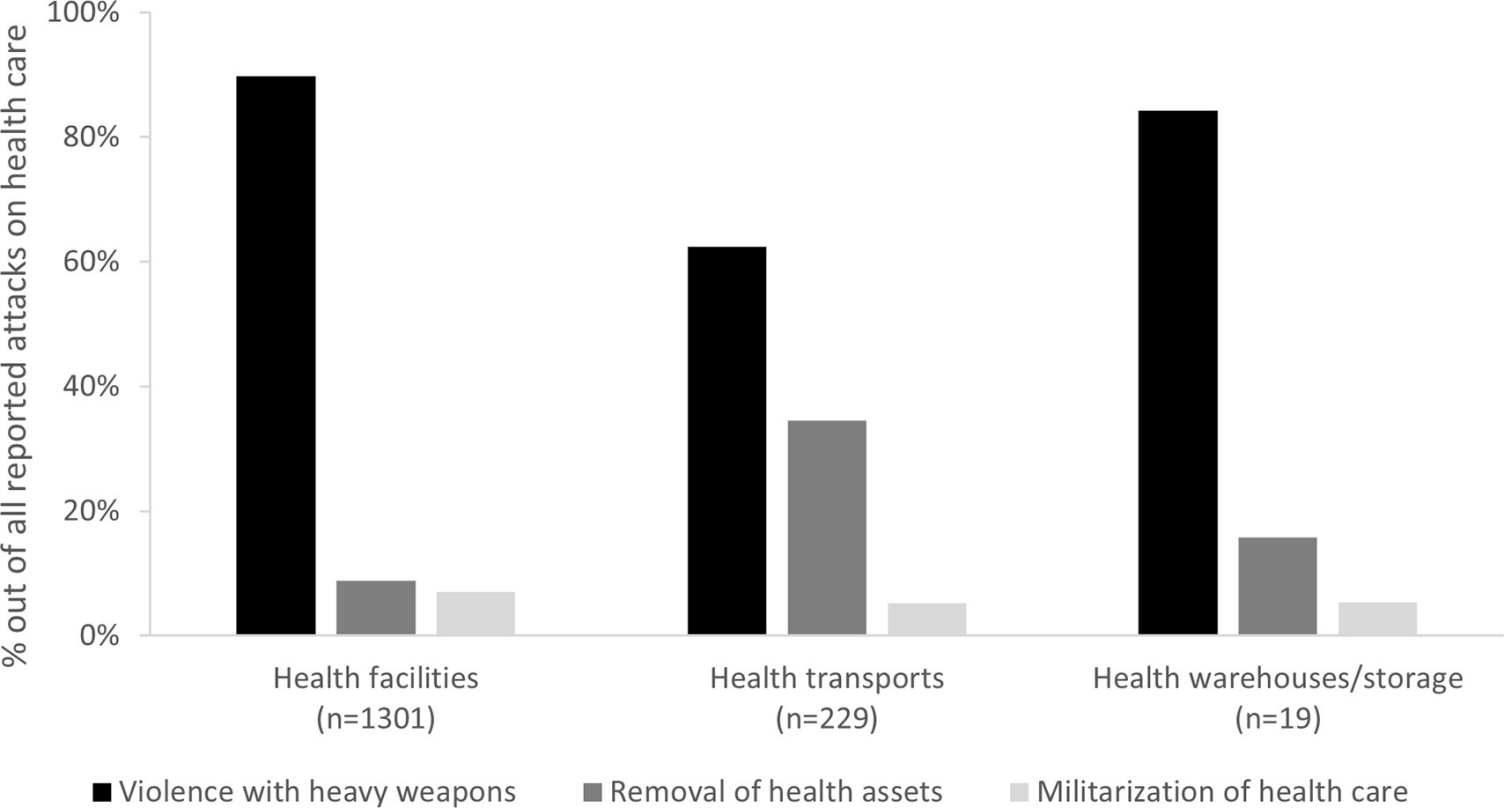
Proportion of types of attacks per affected health infrastructure; as reported to WHO’s Surveillance System for Attacks on Health Care (SSA) in Ukraine, February 2022-August 2023.

**Fig 6:**
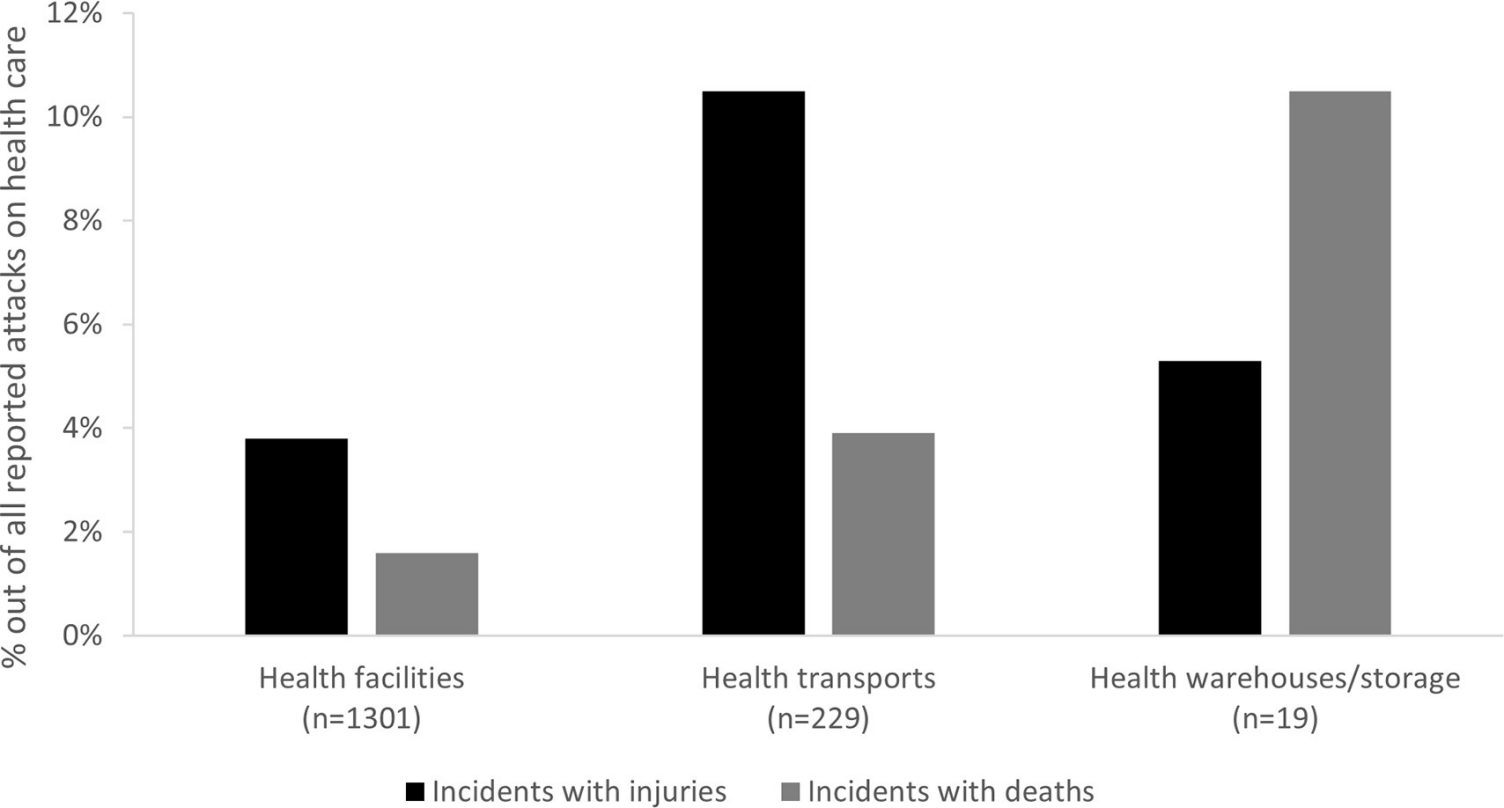
Proportion of attacks resulting in injuries and deaths per affected health infrastructure; as reported to WHO’s Surveillance System for Attacks on Health Care (SSA) in Ukraine, February 2022-August 2023.

Different health resources were confronted with different types of attacks: while heavy weapon attacks were the most common type for all affected health resources, health transports experienced a significantly higher proportion of theft/looting (“removal of assets”) attacks (Fig 5). Casualty patterns also differed: health transports facing attacks had a higher probability of suffering casualties (deaths+injuries) than other health resources (p<0.0001, RR 3.1, 95%CI 1.9–4.9) (Fig 6).

## Discussion

This descriptive analysis of verified attacks on health care in Ukraine reported through the WHO surveillance system highlights the high and sustained burden of attacks in the first 18 months of the war, indicative of the continued lack of respect for IHL by parties to the conflict, despite numerous calls for adherence to IHL and the protection of health services. Reported attacks most frequently consisted of violence with heavy weapons (which typically include artillery and air strike attacks, similar to evidence from e.g. the Syrian conflict and the ongoing war in Gaza [3,12]) and/or more complex typologies, as corroborated by other published datasets [18]. Militarization of health care appeared to represent a particular challenge, as it was often associated with other forms of violence–further research is required to understand whether such militarization is a causal factor of other violence, or rather a consequence. Casualties among patients and health care workers were not homogenously distributed across the different attacks (as also identified in global analyses of attack trends [26]) but tended to occur in a set of high-casualty incidents. Attacks on health transports seemed to carry a higher risk of causing casualties, while health facilities were the most affected infrastructural resource throughout the conflict. While the magnitude of attacks may have diminished following the initial peak in attacks during phase 1, differences in typology of attacks between the different phases of the war were minimal, suggesting the many calls for the protection of health care remained unheard.

The occurrence of a limited number of incidents with a particularly high casualty rate is particularly alarming, and indicates the systematic emphasis that is required on strengthening health preparedness and protection (including early warning systems, efficient evacuation plans that allow for rapid and timely evacuation of staff and patients, and shelters/fortifications). As many incidents, even attacks with heavy weapons (which are typically associated with high casualty rates [3,26]) remained without casualties, we suggest that such protection measures may have been in place in many locations, particularly after phase 1 of the war (when most deaths occurred); however, as long as hostilities persist, health care can only be considered secure if all health services have access to comprehensive protection (including protective measures before, during, and after an attack).

Evidence from other settings confronted with attacks on health care suggests the considerable mental health burden resulting from such violence [27–32]. The burden of high-impact attacks documented here and elsewhere [18], combined with the occurrence of particularly traumatising events such as so-called “double tap” attacks [33], is likely to have contributed considerably to the high rates of absenteeism and health workforce turnover identified in different areas of Ukraine throughout the conflict [34]. Challenges in protecting human resources both during and in the aftermath of attacks (when mental distress may become more apparent) may represent an important barrier for attracting/encouraging the return of the health workforce, as set out as one of the priorities for health system recovery [35].

Attacks on health care represent an important disruptor of health care delivery–the different types of attacks documented here are known to affect the delivery of/access to health care in the long term, including impacting the continuity of long-term/chronic care [13,36,37]. However, attacks are only a part of the wider disruption of the health system due to conflict. Other major risks include attacks on energy infrastructure that affect health care service capacity to provide care [25,38], staff turnover and absenteeism [34], disruptions of supply chains through direct and indirect consequences of conflict [13], and population movements that represent a challenge for the planning and resourcing of health care. These risks translate to a spectrum of difficulties in accessing health care for the population, including increased user costs, increased transportation times, fear of attending health services [37], and a generalised lack of service availability [16]. The specific contribution of attacks on health care to this spectrum of access barriers remains to be elucidated.

As an analysis of publicly available surveillance (SSA) data, this study was not without limitations. First, the passive nature of the data collection that relies on “alerts” of incidents from different sources, initiating the verification process, combined with the complex nature of conflicts can lead to underreporting of attacks, due to difficulties in getting such alerts in e.g. frontline areas confronted with heavy fighting, or due to the normalisation of certain acts of violence that might be considered “not worth reporting”. Particularly in areas where access is near impossible and destruction of civilian infrastructures is nigh complete, such as that reported in e.g. the battle for Bakhmut [39], reporting through the SSA may be rendered quasi impossible. The set of attacks reported on here should thus be considered as a minimum set of verified attacks, rather than an exhaustive overview of attacks occurring in Ukraine. This may also explain the differences in numbers of attacks reported through other channels, using different approaches for incident identification [18,40]. Second, due to security constraints, no spatiotemporal analyses were conducted, as locations of attack incidents are not disclosed through the SSA. Additionally, as attacks incidents did not contain a health service identifier (also for security reasons), analyses at service level–such as the occurrence of repeat attacks–could not be included. Evidence from other datasets from Ukraine suggests that 14% of all attacked facilities faced repeat attacks [18]. Third, the SSA is an incident-based system, and as such is not optimally designed to identify systemic attacks on health care such as structural obstructions to accessing to health care, even though such obstructions can be considered attacks under the WHO definition.

Despite these limitations, this study highlights some key recommendations. First, the volume of attacks reported here and elsewhere–and particularly of violence with heavy weapons [18]–carries important implications for the resources that are needed for reconstruction of damaged or destroyed health resources during conflict, which will need to be anticipated [15,35,41]. Reports of verified attacks can be used to support advocacy on protecting health care and building resilience of health resources, especially in the recovery/reconstruction phase.

Second, the types and volume of attacks in this conflict are indicative of the risks health care continues to face during modern conflict, including most recently the devastating consequences for health care of the conflict in Gaza [42,43], despite the numerous legal frameworks and calls for protection of health care. Awareness on attacks on health care needs to be reinforced and investing resources in preparedness and protection before and during attacks is required–while casualty rates suggest that these may have been in place and functional across many locations in Ukraine, the occurrence of a number of high-casualty incidents represents an important threat to health care. Specific guidance on protecting health transports, which were found to be more vulnerable, may be key in reducing overall casualty numbers. This may be particularly relevant for Ukraine considering the current health care strategies involving the deployment of mobile health units [34,44].

Third, the SSA reports on verified attacks in a standardised manner–its value lies in its systematic collection of data over time. However, underreporting likely occurs, and due to limited interest and changing contexts, attrition of the surveillance quality over time is a risk. Of note, a similar study focusing on the first year of the war and using a different methodology of attack identification and verification found considerable differences in attack numbers, with overall lower numbers being reported using the Berkeley Protocol on Digital Open Source Investigations [18]. These differences are indicative of the challenges in collecting this type of data, and suggest that sustained resourcing of the SSA, as well as use of its data to trigger protection actions, is thus required.

Fourth, this descriptive study of routine programme data opens the door to several operational research questions, which as yet remain unaddressed. Such questions include among others the psychological impacts of specific types of attacks (including repeat attacks and double tap attacks), the economic and health financing impacts of attacks on health care, the associations between attacks on health care and access barriers for the population, the nature and underlying reasons of attacks consisting of militarization of health care, and the good/best practices of protection and mitigation of attacks on health care.

In conclusion, this analysis of public SSA data shows how the number of attacks on health care in Ukraine has been high and sustained over the course of the first 18 months of the war, and how casualties were not homogenously distributed among attack incidents. Health transports were found to be particularly vulnerable. The SSA data from Ukraine demonstrates the crucial role of having prevention, protection, mitigation, and reconstruction strategies in place.
